# A Rare Case of Arytenoid Cartilage Neurofibroma Mimicking Laryngeal Carcinoma

**DOI:** 10.1155/crra/6442641

**Published:** 2026-05-30

**Authors:** Adil Aytaç, Mert Bulut, Deniz Bulut, Bahar Yanık Keyik, Erdoğan Bülbül

**Affiliations:** ^1^ Department of Radiology, Faculty of Medicine, Balıkesir University, Balıkesir, Turkey, balikesir.edu.tr

**Keywords:** arytenoid cartilage, CO₂ laser, laryngeal neurofibroma, magnetic resonance imaging

## Abstract

Neurofibroma is a benign tumor arising from neural crest–derived cells and is characterized by a nonencapsulated growth pattern that incorporates surrounding nerve fibers. Laryngeal involvement is rare, and arytenoid cartilage localization is exceptionally uncommon. We present a 46‐year‐old woman with hoarseness and cough in whom flexible laryngoscopy demonstrated mucosal swelling over the right arytenoid cartilage. Magnetic resonance imaging revealed a 15 × 10 mm lesion that was isointense on T1‐weighted images and heterogeneously hyperintense on T2‐weighted images, with no diffusion restriction and heterogeneous contrast enhancement. Histopathological examination confirmed the diagnosis of neurofibroma. The lesion was completely excised using a CO_2_ laser under microlaryngoscopic guidance. The patient′s symptoms resolved postoperatively, and no recurrence was observed during follow‐up. Although laryngeal neurofibromas lack specific radiological features, magnetic resonance imaging plays a key role in evaluating lesion extent and in supporting the differential diagnosis, particularly by demonstrating the absence of diffusion restriction. To the best of our knowledge, this is the first reported case of an arytenoid cartilage neurofibroma with subglottic extension. Histopathological evaluation remains essential for definitive diagnosis, whereas minimally invasive CO_2_ laser excision represents an effective treatment option for small and localized lesions.

## 1. Introduction

Neurofibroma is a benign tumor arising from neural crest–derived cells, characterized by its nonencapsulated structure that incorporates surrounding nerve fibers [1]. It typically presents as a slowly growing, painless mass, palpable as a soft subcutaneous nodule. Compression may cause tingling, numbness, or pain in the distribution area of the affected nerve [1].

The clinical spectrum of neurofibroma is broad and varies according to the type, number, and location of the tumor, as well as the presence of neurofibromatosis Type 1 (NF1) [2]. Localized neurofibroma is the most common type, usually appearing as a solitary lesion, and generally does not cause clinical problems other than cosmetic concerns [2]. Cutaneous neurofibromas manifest on the skin as multiple small papules or nodules, the number of which increases after puberty in patients with NF1 [2]. Plexiform neurofibroma is a pathognomonic finding of NF1 and carries a risk of malignant transformation [2–4].

Among all benign laryngeal tumors, neurofibromas account for only 0.03%–0.1% [5, 6]. Although 25%–35% of neurofibromas occur in the head and neck region, laryngeal involvement is much rarer [5, 7, 8]. Of 160 reported cases of laryngeal nerve tumors in the literature, only 20 were diagnosed as neurofibroma [5].

Laryngeal neurofibromas most frequently occur in the supraglottic region, particularly at the aryepiglottic fold and epiglottis [5, 6, 9, 10]. Glottic and subglottic involvement of laryngeal neurofibroma is extremely rare, with only about 10 cases reported in the literature [11–15].

Arytenoid cartilage involvement has been reported in only one case [5]. This localization has particular clinical significance due to its direct association with vocal cord movement [9–11]. Neurofibromas at the arytenoid cartilage level may cause symptoms such as dysphonia, phonation disorders, and airway obstruction, and may pose challenges during surgical treatment in terms of both maintaining airway patency and preserving vocal function [9–11].

In this case report, we present an exceptionally rare entity of arytenoid cartilage neurofibroma, previously described in only a few reports in the literature [5, 9–11], with the aim of highlighting its radiological features and differential diagnosis [13–15].

## 2. Case Presentation

**Figure 1:**
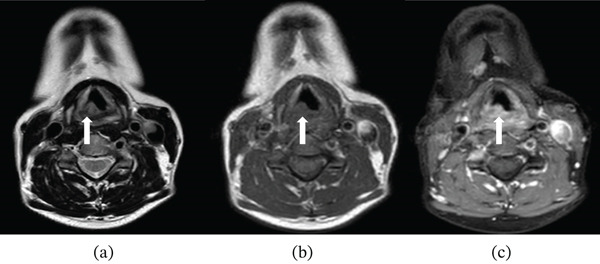
Magnetic resonance imaging of the laryngeal arytenoid neurofibroma. (a) Axial T2‐weighted without fat suppression, (b) axial T1‐weighted without fat suppression, and (c) postcontrast axial T1‐weighted with fat suppression images, with white arrows indicating the lesion.

**Figure 2:**
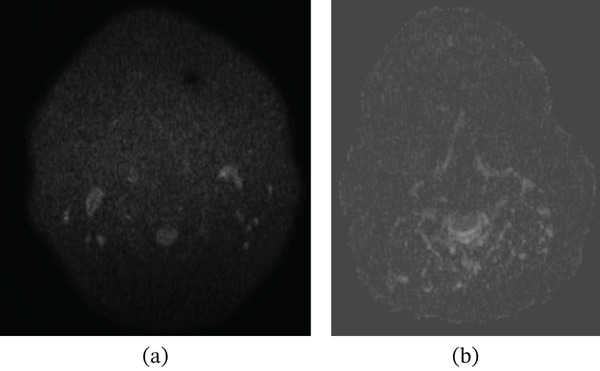
Diffusion‐weighted imaging sections of the arytenoid neurofibroma. (a) Axial B1000 and (b) axial ADC images demonstrate no lesion with diffusion restriction.

A 46‐year‐old female patient presented to the otorhinolaryngology clinic with complaints of hoarseness and cough. No additional symptoms accompanied these complaints. She had a history of 20 pack‐years of smoking and regular alcohol consumption. The patient had no history of chronic illness or regular medication use. Physical examination findings were unremarkable. Endoscopic evaluation revealed bilaterally mobile vocal cords, with mucosal swelling observed in the anterior right arytenoid cartilage. Neck magnetic resonance imaging (MRI), performed due to suspicion of laryngeal carcinoma, demonstrated a 15 × 10 mm lesion located in the posteromedial aspect of the right vocal cord and anterior to the right arytenoid cartilage. The lesion appeared isointense on T1‐weighted images and heterogeneously hyperintense on T2‐weighted images, showed no diffusion restriction on diffusion‐weighted imaging (DWI), and exhibited intense heterogeneous enhancement on postcontrast images (Figures 1 and 2). It extended approximately 5 mm into the subglottic region and 3 mm to the left of the midline, without involvement of the anterior commissure or paralaryngeal fat planes. On T1‐weighted images, no signal alteration of the arytenoid cartilage was observed. Posteriorly, the lesion extended to the anterior border of the hypopharynx without evidence of invasion. Flexible fiberoptic laryngoscopy with incisional biopsy was performed, and histopathological evaluation confirmed the diagnosis of arytenoid cartilage neurofibroma. The lesion was excised using a CO_2_ laser under microlaryngoscopic guidance. No tracheotomy was required, and adequate airway patency was maintained throughout the perioperative period. Flexible laryngoscopic examination demonstrated bilaterally mobile vocal cords, with normal vocal fold abduction and adduction, and no evidence of fixation or impaired movement despite involvement of the arytenoid cartilage. Postoperatively, the patient′s symptoms resolved completely, with no evidence of dysphagia or persistent dysphonia, and normal swallowing and phonatory functions were preserved during follow‐up; no recurrence was detected during 1 year of follow‐up. A comprehensive summary of the radiological findings, perioperative evaluation, surgical management, operative technique, and clinical course of the patient is provided in Table 1.

**Table 1:** Comprehensive summary of radiological findings, operative management, surgical technique, and clinical course of arytenoid cartilage neurofibroma.

Category	Detailed findings and clinical course
Clinical presentation	A 46‐year‐old woman presented with progressive hoarseness and persistent cough. Physical examination findings were otherwise unremarkable.
Endoscopic laryngeal evaluation	Flexible fiberoptic laryngoscopy demonstrated a submucosal swelling localized to the anterior aspect of the right arytenoid cartilage. Bilateral vocal cord mobility was preserved, with normal vocal fold abduction and adduction and no evidence of fixation or impaired movement. The lesion caused partial supraglottic airway narrowing without acute respiratory compromise.
MRI characteristics	Neck MRI revealed a well‐circumscribed 15 × 10 mm lesion centered at the right arytenoid region with approximately 5 mm subglottic extension. The lesion was isointense on T1‐weighted images, heterogeneously hyperintense on T2‐weighted images, and demonstrated heterogeneous postcontrast enhancement without diffusion restriction on DWI.
Anatomical extent	The lesion extended into the subglottic region without invasion of the anterior commissure, paralaryngeal fat planes, or adjacent hypopharyngeal structures. Preservation of surrounding anatomical planes suggested a nonaggressive process.
Preoperative differential diagnosis	Due to the patient′s smoking history, progressive symptoms, arytenoid localization, and subglottic extension, laryngeal malignancy—particularly squamous cell carcinoma—was initially considered. Other differential diagnostic considerations included schwannoma, chondroma, granuloma, and lymphoma.
Radiological features favoring benignity	The absence of diffusion restriction, lack of infiltrative growth, preserved adjacent fat planes, and the absence of cartilage destruction favored a benign neurogenic lesion over malignant laryngeal tumors.
Histopathological diagnosis	Flexible fiberoptic laryngoscopy with incisional biopsy confirmed the diagnosis of arytenoid cartilage neurofibroma.
Surgical planning	Based on the lesion′s limited extent, preserved surrounding anatomical structures, and the absence of extensive cartilage invasion, minimally invasive endoscopic CO_2_ laser excision under microlaryngoscopic guidance was selected to preserve airway patency and phonatory function.
Anesthesia and intraoperative preparation	The procedure was performed under general anesthesia with endotracheal intubation. The patient was positioned for suspension microlaryngoscopy to optimize visualization of the supraglottic and glottic compartments. Airway patency remained stable throughout the operation, and tracheotomy was not required.
Intraoperative findings	Direct suspension microlaryngoscopy demonstrated a smooth‐contoured submucosal mass arising from the anterior aspect of the right arytenoid region with limited subglottic extension. The overlying mucosa was largely preserved, without ulceration or gross infiltrative appearance suggestive of advanced malignancy. No macroscopic invasion of adjacent laryngeal cartilage, anterior commissure, or paraglottic soft tissues was identified.
Surgical technique	The lesion was excised en bloc endoscopically using a CO_2_ laser under microscopic guidance. Stepwise submucosal dissection was performed to achieve complete excision while minimizing thermal injury to adjacent laryngeal mucosa and preserving arytenoid mobility, vocal fold integrity, and surrounding phonatory structures. Particular attention was paid to preservation of the vocal fold complex, arytenoid functional anatomy, and airway continuity throughout the procedure.
Hemostasis and airway management	Intraoperative bleeding was minimal because of the precision and coagulative advantages of the CO_2_ laser technique. Adequate airway patency was maintained throughout surgery without the need for temporary or permanent airway diversion.
Postoperative course	The postoperative period was uneventful. Hoarseness and cough resolved completely, without dysphagia, aspiration, or persistent dysphonia. Normal swallowing and phonatory functions were preserved.
Follow‐up outcome	Follow‐up examinations demonstrated preserved vocal fold mobility and no evidence of residual lesion or local recurrence during 1 year of endoscopic follow‐up.
Educational and clinical significance	This case represents the first reported arytenoid cartilage neurofibroma with subglottic extension and provides a detailed MRI‐based differential diagnostic evaluation together with comprehensive operative management documentation.

## 3. Discussion

Neurofibromas arising from the arytenoid cartilage are of particular clinical relevance because even small lesions at this site can precipitate airway compromise and phonatory dysfunction [9–11]. The present case is noteworthy as the first report of an arytenoid cartilage neurofibroma with subglottic extension. Previous reports further underscore the clinical importance of these tumors: Gupta et al. [9] described an arytenoid laryngeal neurofibroma presenting with dysphonia and dyspnea, with marked symptomatic improvement after surgical excision. Hutnik et al. [10] achieved successful outcomes following emergency surgery in a case presenting with airway obstruction . Son et al. [11] reported a plexiform arytenoid neurofibroma, highlighting the difficulties of preserving vocal function during surgery. In the series reported by Naidu et al. [5], only one case originated from the arytenoid cartilage. Collectively, these examples demonstrate that even small arytenoid cartilage neurofibromas can cause significant functional impairment and necessitate meticulous surgical planning. In this case, the initial suspicion of laryngeal carcinoma was raised by the patient′s progressive symptoms and the endoscopic finding of a firm submucosal mass with subglottic extension. Given that squamous cell carcinoma is the most common laryngeal malignancy and can occasionally present with submucosal growth, excluding malignancy was the primary clinical priority.

Imaging modalities, particularly MRI, play a critical role in assessing the size, extent, and relationships of the lesion with adjacent structures [16–18]. Neurofibromas typically appear iso‐ to hypointense on T1‐weighted images, heterogeneously hyperintense on T2‐weighted images, and show heterogeneous enhancement after contrast administration; occasionally, a “target sign” may be observed, although this finding is not specific, as it may also occur in schwannomas [17, 18]. Notably, prior case reports have not mentioned DWI features. In our case, the absence of diffusion restriction on DWI emerged as an important finding, reducing the likelihood of malignancy. This detail may enhance the role of MRI in differentiating laryngeal neurofibromas from malignant lesions.

CT and MRI play complementary roles in the evaluation of laryngeal tumors. CT is particularly advantageous for assessing cortical cartilage destruction, calcification, ossification, and chondroid matrix mineralization, which may aid in the evaluation of cartilaginous tumors and invasive laryngeal malignancies. In contrast, MRI offers superior soft‐tissue contrast resolution and is more effective in evaluating submucosal tumor extent, paraglottic soft tissues, lesion margins, and relationships with adjacent anatomical structures. In the present case, MRI was preferred because the lesion demonstrated predominantly submucosal soft‐tissue characteristics without evidence of extensive cartilage destruction. Although additional CT imaging was recommended by the radiologist to further evaluate possible subtle cartilage involvement and mineralization, the referring clinician considered MRI findings sufficient for preoperative assessment and surgical planning; therefore, CT examination was not performed.

Although the imaging findings are not pathognomonic, this case demonstrates several features of radiological relevance. The submucosal localization at the arytenoid cartilage with extension into the subglottic region represents an exceptionally rare anatomical distribution. In addition, the absence of diffusion restriction on DWI may serve as a supportive feature favoring a benign etiology and may aid in differentiating neurofibroma from malignant laryngeal lesions. Therefore, the radiological value of this case lies in the integration of imaging characteristics, anatomical localization, and differential diagnostic considerations rather than in a single distinctive imaging feature.

The radiological contribution of the present case lies not in a pathognomonic imaging feature but rather in the integrated interpretation of lesion morphology, anatomical localization, preserved surrounding anatomical planes, and DWI findings in the differential diagnosis of rare submucosal laryngeal tumors. These features may support a more cautious radiological interpretation when evaluating submucosal arytenoid lesions that clinically mimic laryngeal malignancy.

Radiological differential diagnosis plays a crucial role in preventing misinterpretation of submucosal laryngeal lesions. Schwannoma is the most important benign mimic and may be difficult to distinguish from neurofibroma on routine MRI; however, schwannomas are typically well‐encapsulated and sharply marginated, whereas neurofibromas are characteristically unencapsulated and may appear more infiltrative [17]. Other benign lesions, including granulomas and mucus retention cysts, generally lack the solid soft‐tissue characteristics of neurogenic tumors. Chondromas are identifiable by cartilaginous calcifications on CT, lipomas demonstrate uniform fat signal intensity, and hemangiomas are characterized by serpiginous vascular channels and flow voids on MRI [13–15]. Among malignant lesions, squamous cell carcinoma is most frequently considered; it usually exhibits diffusion restriction and irregular invasive margins, features that were absent in our case. Chondrosarcomas are often associated with a calcified cartilaginous matrix and bone destruction, whereas lymphomas typically present as homogeneously enhancing, diffusion‐restricting submucosal masses. Infectious and inflammatory conditions such as tuberculosis and sarcoidosis may mimic neoplasms but generally display distinct enhancement patterns and are accompanied by systemic manifestations [14, 15]. Although histopathological confirmation remains the diagnostic gold standard, radiological findings provide critical guidance for refining the differential diagnosis.

Surgical intervention is the mainstay of treatment. For small and localized lesions, endoscopic resection under microlaryngoscopy is the most appropriate approach [19, 20]. CO_2_ laser excision offers particular advantages at the arytenoid level due to its minimally invasive nature, reduced bleeding, and improved visualization [19, 20]. For larger lesions or those extending to adjacent structures, open surgical approaches may be required [21]. Total laryngectomy is considered only for very extensive disease or when malignancy is suspected [21]. In patients with airway obstruction or anticipated intraoperative complications, tracheostomy may be necessary. Postoperative follow‐up is essential to assess recurrence risk and monitor functional outcomes. Although neurofibromas are benign, their nonencapsulated nature may lead to recurrence if complete excision is not achieved. Therefore, annual endoscopic follow‐up is recommended, particularly for arytenoid cartilage neurofibromas. In our case, the small lesion was completely excised with CO_2_ laser under microlaryngoscopy, achieving full resection while preserving vocal function [19, 20].

A limitation of this case is the lack of archived preoperative and postoperative endoscopic photographic documentation. Another limitation is the absence of CT imaging for assessment of cartilage involvement and lesion characterization.

This case represents the first reported arytenoid cartilage neurofibroma with subglottic extension. As laryngeal neurofibromas do not exhibit pathognomonic radiological features, histopathological confirmation remains the diagnostic cornerstone. For small lesions, minimally invasive CO_2_ laser surgery offers an effective therapeutic approach, simultaneously ensuring airway safety and preserving vocal function.

## Author Contributions

All of the authors declare that they have all participated in the design, execution, and analysis of the paper.

## Funding

No funding was received for this manuscript

## Disclosure

All authors have approved the final version of the manuscript.

## Consent

Written informed consent was obtained from the patient for publication of this case report and any accompanying images.

## Conflicts of Interest

The authors declare no conflicts of interest.

## Data Availability

The data that support the findings of this study are available on request from the corresponding author. The data are not publicly available due to privacy or ethical restrictions.
